# Binding of Platinum(II) to Some Biologicaly Important Thiols

**DOI:** 10.1155/MBD.1999.355

**Published:** 1999

**Authors:** Biljana V. Petrovic, Milos I. Djuran, Zivadin D. Bugarcic

**Affiliations:** University of Kragujevac Faculty of Science Department of Chemistry R. Domanovica 12, P. O. Box 60 Kragujevac YU-34 000 Serbia

## Abstract

The reactions between [Pt(terpy)Cl^+^ and thiols, such as glutathione, L-cysteine, D-penicillamine and thioglycolic acid have been Studied by conventional UV-VIS spectrophotometry and H NMR spectroscopy. The second-ordero rate constants, K_2_, are similar for these four thiols, varying between 1.06 x 10^-2^ and 6.10 x 10^+3^ M^-1^ s^-1^ at 25^°^C. The activation entropies have large negative values between -100 and -200 J mol^-1^ which are compatible with an associative A mechanism. However, L-methionine, as thioether ligand, is unreactive under the same experimental conditions. The obtained results have been analyzed in relation to the antitumor activity and toxicity of platinum(II) complexes.


### BINDING OF PLATINUM(II) TO SOME BIOLOGICALY IMPORTANT THIOLS

Biljana V. Petrovic, Milos I. Djuran and Zivadin D. Bugarcic*

University of Kragujevac, Faculty of Science, Department of Chemistry,
R. Domanovica 12, P. O. Box 60, YU-34 000 Kragujevac, Yugoslavia
e-mail: bugi@knez.uis.kg.ac.yu

**Abstract**

The reactions between $[Pt(terpy)Cl^{+}$ and thiols, such as glutathione, L-cysteine, D-penicillamine and thioglycolic acid have been studied by conventional UV-VIS spectrophotometry and $^{1}H$ NMR spectroscopy. The second-order rate constants, $k_2$, are similar for these four thiols, varying between $1.06 \times 10^{-2}$ and $6.10 \times 10^{-3}$ $M^{-1}s^{-1}$ at 25°C. The activation entropies have large negative values between -100 and -200 J $mol^{-1}$ which are compatible with an associative A mechanism. However, L-methionine, as thioether ligand, is unreactive under the same experimental conditions. The obtained results have been analyzed in relation to the antitumor activity and toxicity of platinum(II) complexes.

**Introduction**

The study of the aqueous chemistry of platinum(II) complexes has been extensively investigated over the past few decades especially due to its relevance to anticancer activity[1-3]. For instance, *cis*-diamminedichloroplatinum(II), *cis*-$[Pt(NH_3)_2Cl_2]$, tradenamed cisplatin, has become one of the most widely used antitumor compound in oncology. On the other hand, this use has been limited by toxicity to the kidney and other side effects (nausea, vomiting, neurotoxicity, *etc.*) [4,5]. The binding of the platinum(II) to protein-bound sulfhydryl (thiol) group (-SH) is thought to be responsible for the observed toxic side effects [6]. However, nephrotoxicity is supposed to be the result of the inactivation of certain enzymes due to the binding of cisplatin to the sulfhydryl groups of cysteine residues [7]. Moreover, sulfur appearing as the sulfhydryl residue of the essential amino acid L-cysteine, as an excellent nucleophile for platinum(II).

The non-antitumor active monofunctional complex, $[Pt(terpy)Cl]^{+}$, chloro(2,2':6',2''-terpyridine)-platinum(II), shown below, is a very useful substrate for kinetic studies on substitution reaction of square-planar $d^{8}$ coordination compounds. In spite of that, the number of kinetic studies of $[Pt(terpy)Cl]^{+}$ is small [8-12].

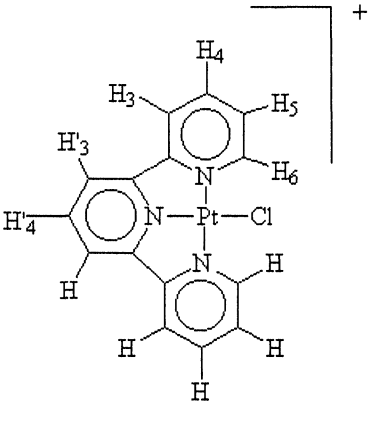

On the other hand, the analogous $[Pt(dien)Cl]^{+}$ (dien = 1,5-diamino-3-azapentane) has been extensively investigated. $[Pt(terpy)Cl]^{+}$ reacts $10^{3}$ - $10^{4}$ times faster than $[Pt(dien)Cl]^{+}$ [8,9].

We recently reported a detailed kinetic and mechanistic study of the complex formation between different platinum(II) complexes and some thiols [10,12,13]. Since interactions between platinum(II) complexes and sulfur-containing ligands are very important from biological and medicinal aspects, in the present paper, we reported a study of the rection between $[Pt(terpy)Cl]^{+}$ and some biologically important thiols such as: glutathione, L-cysteine, D-penicillamine and thioglycolic acid.

Chart 1.

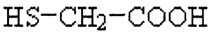

Thioglycolic acid

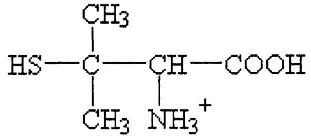

D-Penicillamine

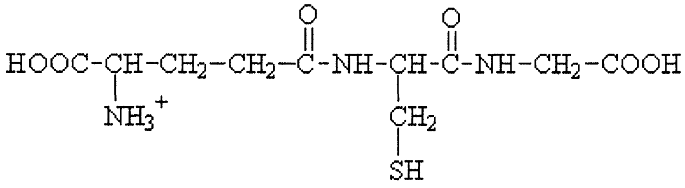

Glutathione

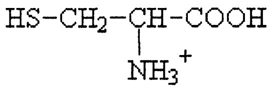

L-Cysteine

Complex formation between $[Pt(terpy)Cl]^+$ and those thiols have been studied as a function of temperature (288 - 308 K) by the use of conventional UV-VIS spectrophotometry and $^1H$ NMR spectroscopy.

### Materials and Methods

### *Chemical and Solutions*

The complex chloro(2,2':6',2''-terpyridine)platinum(II)chloride dihydrate, [Pt(terpy)Cl]Cl x 2 $H_2O_1$ was prepared according to published methods [14] and characterized by elemental analysis, UV-VIS and H NMR spectra. Deuterated methanol, $d_4$-$CD_3OD$, deuterium chloride, $d_1$-DCl (Aldrich Chemical Co.), thioglycolic acid (Fluka, 98%), L-cysteine, L-methionine, glutathione and D-penicillamine (Sigma Chemical Co.) were used as received.

### *NMR measurements*

$^1$NMR spectra were obtained with a Varian Gemini 200 MHz spectrometer equipped with a 5 mm probe for H NMR measurements. Equimolar amounts of the platinum(II) complex and thiol ligands were mixed in an NMR tube. The final solution was 10 mM in each reactant. The internal reference was TSP (sodium trimethylsilylpropane-3-sulfonate). A drop of DCl was added to the ligand solution to prevent its deprotolysis and hydrolysis of the complex. After mixing reactant solutions, the spectra were immediately recorded as a function of time. The reactions have been studied at 22$^o$C in $d_4$-$CD_3OD$ as a solvent.

### *Kinetic measurements*

The reactions were sufficiently slow to be followed spectrophotometrically by measuring the change in absorbance at suitable wavelengths as a function of time. The kinetics were followed using a Varian Super-Scan 3 double-beam spectrophotometer equipped with water thermostatted cells. The reactions were iniated by adding 0.3 ml of a thermostatted solution of the platinum complex to 2.5 ml of a solution of the nucleophile in the thermostatted spectrophotometric cell. After preliminary repetitive scan experiments in the range 260-400 nm to search for isobestic points and spectral changes, the kinetics were studied by measuring the change in absorbance at suitable wavelengths (340, 345 or 350 nm) as a function of time. The kinetic measurements were performed between 288 and 308 K using a large excess of ligands (at least ten-fold excess). The pseudo-first-order rate constants ($k_{obsd}$, $s^{-1}$) have been obtained graphically from a plot of $\ln(A_\infty - A_t)$ *vs.* time [15] ($A_t$ and $A_\infty$ are the absorbances of the reaction mixture at time *t* and at the end of the reaction, respectively, usually after 10 half-lives) or from a non-linear least-squares fit of the experimental data to Eq. 1. with $A_0$, $A_\infty$ and $k_{obsd}$ as the parameters to be optimized. Rate constants were accurate within 5%.

$$A_t = A_\infty + (A_0 - A_\infty) \exp(-k_{obsd}\, t) \qquad (1)$$

Methanol/water solutions (95/5%,v/v) were used and the ionic strength and the acidity of the solutions were adjusted to 0.10 M with methansulphonic acid, $CH_3SO_3H$ (Aldrich, 99%).

**Results and Discussion**

The spectrum of the reaction mixtures evolves in all cases with time in a first order fashion and with well maintained isosbestic points, from that of the chloro substrate $[Pt(terpy)Cl]^+$ to that of an authentic sample of the substituted products $[Pt(terpy)(SR)]^+$ measured under the same experimental conditions, clearly indicating that the process studied is the displacement of the coordinated chloride by the thiol.

Previous studies of $[Pt(terpy)Cl]^+$ mainly focused on its interaction with biological molecules like DNA, nucleic acids and proteins [16-18]. Initial studies of the binding of $[Pt(terpy)Cl]^+$ to cell thymus DNA revealed covalent interactions with the bases, as well as intercalation [16]. The platinum(II) terpyridine complex has a tendency to aggregate in aqueous solution and the dimerization constant being (4±2) x 10 $M^-$ [16]. However, hydrolysis of the chloro ligand from the complex is relatively fast (ca. $10^{-2}$ $s^{-1}$) and $[Pt(terpy)Cl]^+$ exists in aqueous solution [12]. In order to prevent dimerization and hydrolysis of the complex all reactions were studied in acidic methanol/water (95/5 %,v/v) solution. Under these conditions the complex is stable [12].

The proton NMR spectrum of the $[Pt(terpy)Cl]^+$ is in accordance with those previously published [16]. The doublet furthest downfield in the spectrum of [Pt(terpy)Cl] is assigned as $H_6$. The chemical shifts of these resonances are approximately constant among the thiol complexes studied, and appear ca. 0.40 ppm further downfield in the spectrum. Difference in the H NMR chemical shifts of $H_6$ proton for [Pt(terpy)Cl] and [Pt(terpy)(SR)] complexes, Table I., have been used to follow reactions between the platinum(II) complex and thiol ligands.

**Table I.** Proton Nuclear Magnetic Resonance Chemical Shifts of coordinated and non-coordinated Thiols in $d_4$-$CD_3OD$, according to $H_6$ protons from $[Pt(terpy)Cl]^+$.

| Ligand | $\delta_{coord.}$ /ppm | $\delta_{non\text{-}coord.}$ /ppm |
|---|---|---|
| D-Penicillamine | 9.78 | 9.08 |
| L-Cysteine | 9.45 | 9.08 |
| Thioglycolic acid | 9.55 | 9.08 |
| Glutathione | insoluble in $d_4$-$CD_3OD$ | |

The data were obtained by monitoring the changes in the $^1$H NMR spectra as a function of time. The reaction between [Pt(terpy)Cl] and thiols was followed by H NMR spectroscopy and the first change occurring in the spectrum (observable within ca. 5-8 min. of mixing) is the growth of the doublet at 9.45 ppm for L-cysteine, for instance. These signals are assignable to the $H_6$ proton of product, [Pt(terpy)(SR)] and their intensities were compared with those of the signal at 9.08 ppm corresponding to the $H_6$ proton of $[Pt(terpy)Cl]^+$. The doublet due to [Pt(terpy)Cl] decreased in intensity with time, while the other peaks, due to [Pt(terpy)(SR)] increased. The values of the rate constants were determined by fitting the data from the early part of the reaction (up to 1h) to a second-order process [15]. *i.e.* by plotting $x/a_0(a_0-x)$ *vs.* time, $t$, ($a_0$ = initial concentration of [Pt(terpy)Cl] and $x$ = concentration of [Pt(terpy)(SR)] at time $t$).

The observed pseudo-first-order rate constants from the conventional kinetic experiments were obtained from a linear least square-analysis of the first three half-lives and represent the average of two to three experiments. Figure 1 shows the concentration dependence of $k_{obsd}$.

The rate constants are linearly dependent of the concentration of excess of thiols. The second-order rate constants $k_2$ for formation of the complex were derived by fitting the $k_{obsd}$ values to Eq. 2 according to the two-terms rate law for nucleophilic substitutions at $d^8$ metal complexes:

$$k_{obsd} = k_1 + k_2\,[\mathrm{Thiol}] \qquad (2)$$

One reaction involves direct nucleophilic attack by entering ligand ($k_2$), the other reaction involves the rate determining formation of a solvent ligand complex ($k_1$) [19].

The second-order rate constants, $k_2$, obtained by conventional UV-VIS spectrophotometry and $^1$H NMR spectroscopy are summarised in Table II.

From this table can be concluded that these thiols are very good entering groups for the platinum(II) complex. However, the variation in size, bulkiness and solvation of the entering thiols reflect to their properties as nucleophiles. The reaction of the Pt(II) complex and D-pencillamine is the slowest one. This could be explained by the steric effect of the two methyl groups on the neighbouring carbon to the sulphur atom (Chart 1).

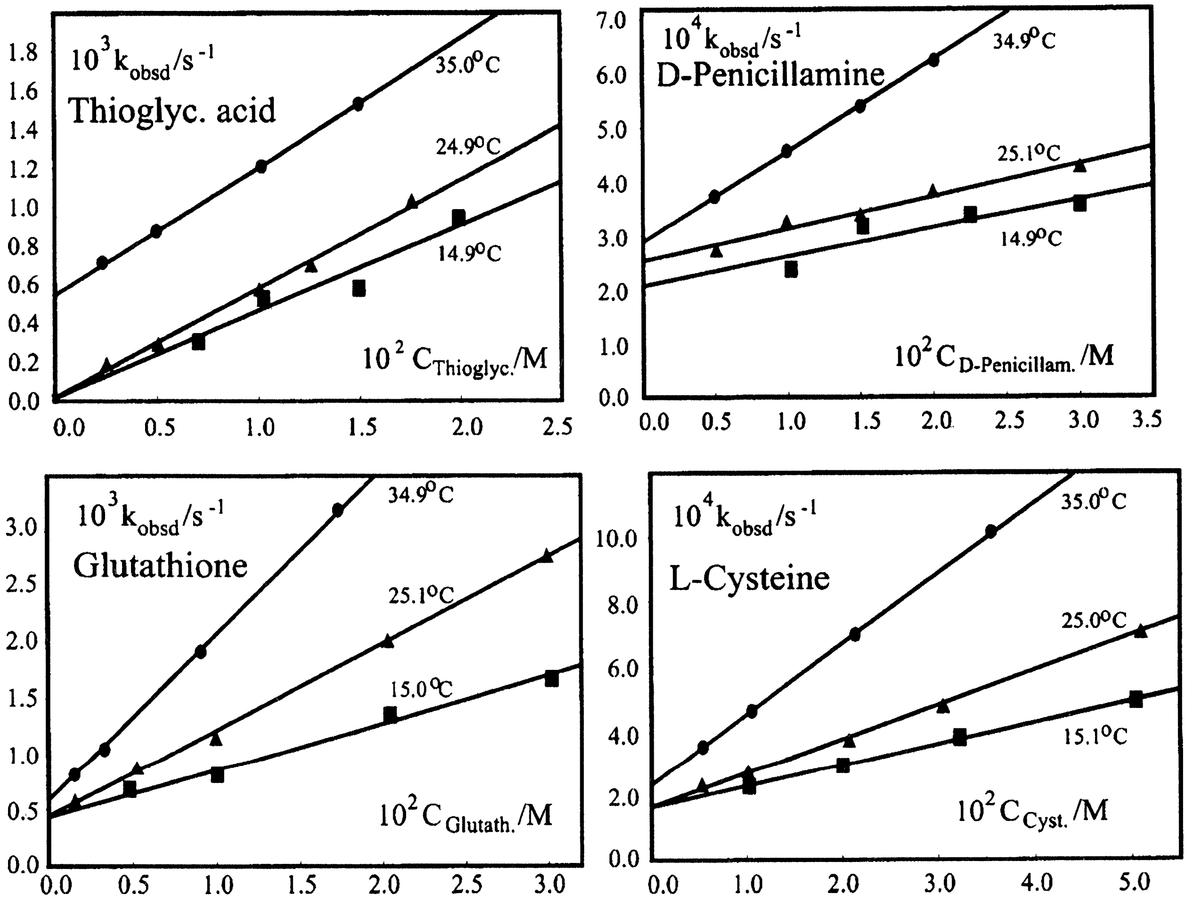

Figure 1. Observed pseudo-first-order rate constants as a function of excess of thiols.

**Table II.** Rate Constants and Activation Parameters for the Reactions between $[Pt(terpy)Cl]^+$ and some Thiols.

| **Ligand** | **$10^2$ $k_2$ /$M^{-1}s^{-1}$** | **$10^2$ $k_2^a$ /$M^{-1}s^{-1}$** | **$10^4$ $k_1$ /$s^{-1}$** | **$\Delta H_2^{\neq}$ / $KJmol^{-1}$** | **$\Delta S_2^{\neq}$ / $Jmol^{-1}$** |
|---|---|---|---|---|---|
| L-Cysteine | 1.06 ± 0.2 | 4.90 ± 0.6 | 1.65 ± 0.8 | 47 ± 4 | -114 ± 5 |
| Glutathione[b] | 7.77 ± 0.8 | -//- | 4.42 ± 0.8 | 44 ± 3 | -110 ± 6 |
| Thioglyc. acid | 5.62 ± 0.2 | 5.20 ± 0.4 | 1.71 ± 0.2 | 15 ± 2 | -216 ± 4 |
| D-Penicillamine | 0.61 ± 0.01 | 0.18 ± 0.01 | 2.52 ± 0.4 | 39 ± 5 | -145 ± 4 |

[a]The rate constant obtained by $^1H$ NMR spectroscopy at 22°C.
[b]Glutathion is unsoluble in $d_4$-methanol.

Moreover, stronger steric interactions between the bound terpy and the entering D-pencillamine are expected. At the same time glutathione is considerable more reactive than expected. This anomaly seems to suggest an appreciable anchimeric effect capable to reduce the activation free energy of the substitution, arising from hydrogen bonding interactions between the acidic group located in a suitable position of the nucleophile and the developing chloride in the transition state. The $k_1$ term, obtained as intercepts of the plots of $k_{obsd}$ *vs.* thiol concentrations, is small, ca. $10^{-4}$, contributes little to the observed rates and is independent of the nature of the thiols.

The rate constants obtained at the three temperatures allow the calculation of the corresponding enthalpies and entropies of activation through a fit to the Eyring equation [15].

The activation entropies listed in Table II have large negative values that are compatible with an associative A mechanism.

Undissociated thiols, HSR, may be expected to behave like thioethers, $RSR_1$, taking into account the different electronic and steric peculiarities of the radical H as compared to $R_1$. The nucleophilicity of thioethers towards square-planar tetra-coordinate Pt(II) and Pd(II) complexes has been proved [20] to be sensitive to both electronic and steric effects. However, L-methionine, as thioether ligand, seems to be unreactive under the same experimental conditions and the spectrum of the [Pt(terpy)Cl] remains unchanged within several hours even in the presence of a large excess of ligand. The difference can be explained by the steric effects of the [Pt(terpy)Cl] and of the thioethers, but this cannot be the main reason. Moreover, it has been prepared [Pt(terpy)(tx)]$(CF_3SO_3)_2$ complex (tx = 1,4-thioxane) which is relatively stable in the solid state, but in the solution has been observed solvolyzes [21].

Since the thiols are undissociated in these experiments, this means that the act of substitution is immediately followed by deprotonation of the products [Pt(terpy)(RSH)]$^{2+}$ and the reaction leads to the thiolate complex [Pt(terpy)(SR)] which is the final product. In the reaction with L-methionine there is no possibility for the deprotonation and this could be the main reason for the difference in the reactivity between thiols and thioethers with [Pt(terpy)Cl]. It seems, therefore, that thioethers do not react just because the resulting bis-cationic product cannot be stabilized by deprotonation [12]. Therefore, even if there is not any difference in the nucleophilicity of thiols and thioethers, a difference is clearly evident in the relative stability of the reaction products [12].

By comparison, from Table III, can be seen that [Pt(terpy)Cl]$^+$ is more reactive than [Pt(dien)Cl]$^+$ in the reactions with these thiols [22].

**Table III.** The second-order rate constants for the reactions between thiols and platinum(II) complexes.

| **Ligand** | **$k_2$ / $M^{-1}s^{-1}$ [Pt(dien)Cl]$^+$** | **$k_2$ / $M^{-1}s^{-1}$ [Pt(terpy)Cl]$^+$** |
|---|---|---|
| Thiglycolic acid | $7.86 \times 10^{-3}$ | $5.62 \times 10^{-2}$ |
| L-Cysteine | $1.43 \times 10^{-3}$ | $1.06 \times 10^{-2}$ |
| D-penicillamine | $8.04 \times 10^{-4}$ | $6.02 \times 10^{-3}$ |
| Glutathione | $3.85 \times 10^{-3}$ | $7.77 \times 10^{-2}$ |

This could be explained by the peculiar coordination geometry of the aromatic pyridine ring. The complex [Pt(terpy)Cl] appears to be more reactive than [Pt(dien)Cl] in accordance with the greater possibility of $\pi$-interaction and $\pi$-*trans* effect probably operating as well. This could be supported by the results of the crystal structure analysis of the [Pt(terpy)Cl] complex [23]. The Pt-N distance to the middle nitrogen atom of the terpyridine ligand, 1.930(4) Å is slightly shorter than the distances of Pt to the other two nitrogen atoms, N1, 2.018(5) and N3 2.030(5) Å [23]. On the other hand, in the present study the terpy complex reacts only *ca.* 10 times faster than dien complex. This small difference in the reactivity of these two complexes could be explain by less nucleophilicity of the entering thiols to the terpy complex. Moreover, stronger steric interactions between the bound terpy and the entering thiols could be expected.

However, the present results explicitly demonstrate that sulfur-containing biomolecules are very reactive species and they have a high affinity for the Pt(II) complex, Table II. Moreover, they may react with Pt(II) before the complex can reach DNA. Certainly, intercellular thiol peptides, such as glutathione and metallothionine, may react with antitumor Pt(II) complexes thus preventing or reducing the amount of platinum binding to DNA. Reaction with SH groups of protein side chains (*e.g.*, in metallothionein and glutathione) is thought to trap and deactivate the drug before it reaches its cellular target DNA to form the 1,2-intrastrand cross-link of guanine bases, the likely cytotoxic adduct. It means that the bond between the Pt(II) complex and sulfur-containing molecules is rather strong making and it is very difficult to remove Pt(II) [24]. The large stability of the Pt(II) complexes with the sulfur biomolecules is thought to be responsible for the observed toxic side effects [7]. Supportive for this mechanism is that the total protein-bound sulfhydryl groups is depleted (14%) in kidneys after cisplatin admistration, especially in the mytochonrial fraction [25].

*Acknowledgement.* This work was supported by the Ministry of Science and Technology of the Republic of Serbia (Grant No. 02E35). Our thanks due to Miss Biljana Mojsilovic for assistance with recording H NMR spectra.

**References**

[1] *Platinum and Other Metal Coordination Compounds in Cancer Chemotherapy 2,* Pinedo, H. M.;Schornagel, J. H., Eds.; Plenum, New York, 1996.
[2] Reedijk, J., *Chem. Commun.,*1996, 801.
[3] *Bioinorganic Chemistry,* Bertini, I.;Gray, H. B.; Lippard, S. J.; Valentine, J. S. Eds.; University Science Books, Mill Valley, CA, 1994.
[4] Krakoff , I.H. *Cancer Treat. Rep.*, 1979, **63,** 1523 - 1525.
[5] Corden, B.J. *Inorg. Chim. Acta,* 1987, **137,** 125 - 130.
[6] Borch, R. F.; Katz, J.C.; Lieder, P.H.; Pleasants, M. E. *Proc. Natl. Acad. Sci. U. S. A.* 1980, **77**, 5441 - 5444.
[7] Borch, R. F.; Pleasants, M. E. *Proc. Natl. Acad. Sci. U. S. A.* 1979, **76**, 6611 - 6614.
[8] Mureinik, R. J.; Bidani, M. *Inorg. Chim. Acta* 1978, **29**, 37 - 41.
[9] Pitteri, B.; Marangoni, G.; Cattalini, L.; Bobbo, T. *J. Chem. Soc. Dalton Trans.* 1995, 3853 - 3859.
[10] Bugarcic, Z.D.; Djordjevic, B.; Djuran, M.I. *J. Serb. Chem. Soc.* 1997, **62**, 1031-1036
[11] Pitteri, B.; Marangoni, G.; Viseutim, F. V.; Cattalini, L; Bobbo, T. *Polyhedron* 1998, **17**, 475-482.
[12] Annibale, G.; Brandolisio, M.; Bugarcic, Z. D.; Cattalini, L. *Trans. Met. Chem.* 1998, **23**, 715-720.
[13] Bugarcic, Z.D.; Djordjevic, B.V. *Chem. Monthly* 1998, **129**, 1267-1274.
[14] Annibale G., Brandolisio M., Pitteri B. *Polyhedron* 1995, **14**, 451-453.
[15] Espenson, J.H. Chemical Kinetics and Reaction Mechanisms, 2nd ed. McGraw-Hill, New York, 1995, chapter 2 and 6.
[16] Jennette, K.W.; Gill, J.T.; Sadownick, J.A.; Lippard, S. *J. Am. Chem. Soc.* 1976, **98**, 6159 – 6168.
[17] Constable, E. C. *Adv. Inorg. Chem. Radiochem.*1986, **30**, 69 - 72.
[18] Ratilla, E.M.A.; Brothers, H.M.; Kostic, N.M. *J. Am. Chem. Soc.*1987, **109**, 4592 - 4598.
[19] Kotowski, M.; van Eldik, R. In *Inorganic High Pressure Chemistry: Kinetics and Mechanism*, van Eldik R., Ed.; Elsever, Amsterdam, 1986; chapter 4.
[20] Elmroth, S.; Bugarcic, Z. D.; Elding, L. I. *Inorg. Chem.* 1992, **31**, 3551 - 3554.
[21] Bugarcic, Z. D.; Annibale, G.; Cattalini, L., *unpublished results*
[22] Bugarcic, Z. D.; Djordjevic, B.V. and Djuran, M. I. *submited to Polyhedron*
[23] Yip, H.K.; Cheng,; L.K., Cheung, K. K.; Che, C. M. *J. Chem. Soc. Dalton Trans.* 1993, 2933 - 2938.
[24] Bugarcic, Z. D.; Leovac, V. M. and Baltic, V. *Arch. Of Oncol.* 1997, **5**, 183-185.
[25] Singh, G. *Toxicology* 1989, **58**, 71-73.

**Received: September 20, 1999 - Accepted: October 1, 1999 - Received in revised camera-ready format: October 6, 1999**

